# Supplementary material for: CaBP1 and 2 enable sustained CaV1.3 calcium currents and synaptic transmission in inner hair cells
Source: eLife. 2024 Dec 24;13:RP93646. doi: 10.7554/eLife.93646 (PMC11668525; doi:10.7554/eLife.93646)
Supplement: Supplementary file 1. — The table lists the adjusted p-values of the Tukey’s multicomparisons tests upon one-way (20 Hz clicks) or two-way ANOVA (otherwise) for the ABR thresholds and the click-evoked ABR wave I–V latencies. Very low p-values are represented by asterisks: p < 0.001 (***), p < 0.0001 (****). [file elife-93646-supp1.docx]

| *­­­* | | *Cabp2*-injected DKO animals vs control DKO  animals | DKO vs WT  animals | *Cabp2*-injected DKO animals vs WT animals |
| --- | --- | --- | --- | --- |
| **ABR thresholds** | 6 kHz tone burst | **** | **** | 0.4 |
|  | 12 kHz tone burst | **** | **** | 0.002 |
|  | 24 kHz tone burst | **** | **** | **** |
|  | 20-Hz click | **** | **** | < 0.03 |
| **ABR wave latencies** | Wave I | **** | **** | **** |
|  | Wave II | 0.1 | **** | **** |
|  | Wave III | < 0.03 | *** | **** |
|  | Wave IV | 0.1 | **** | **** |
|  | Wave V | 0.9 | **** | **** |

**Supplementary Table 1.** **Statistical analysis of ABR thresholds and ABR wave I-V latencies**. The table lists the adjusted *p*-values of the Tukey’s multicomparisons tests upon one-way (20-Hz clicks) or two-way ANOVA (otherwise) for the ABR thresholds and the click-evoked ABR wave I-V latencies. Very low *p*-values are represented by asterisks: *p* < 0.001 (***), *p* < 0.0001 (****).
